# Coupling mechanisms coordinating mRNA translation with stages of the mRNA lifecycle

**DOI:** 10.1080/15476286.2025.2483001

**Published:** 2025-03-21

**Authors:** Valeria Famà, Lucia Coscujuela Tarrero, Roberto Albanese, Lorenzo Calviello, Stefano Biffo, Mattia Pelizzola, Mattia Furlan

**Affiliations:** a Center for Genomic Science of IIT@SEMM, Istituto Italiano di Tecnologia (IIT), Milan, Italy; b Department of Oncology and Emato-Oncology, University of Milan, Milan, Italy; c Human Technopole, Milan, Italy; d National Institute of Molecular Genetics, Fondazione Romeo ed Enrica Invernizzi, INGM, Milan, Italy; e Department of Biosciences, University of Milan, Milan, Italy; f Department of Biotechnology and Biosciences, University of Milano-Bicocca, Milan, Italy

**Keywords:** Translation, transcription, processing, export, decay, coupling

## Abstract

Gene expression involves a series of consequential processes, beginning with mRNA synthesis and culminating in translation. Traditionally studied as a linear sequence of events, recent findings challenge this perspective, revealing coupling mechanisms that coordinate key steps of gene expression, even when spatially and temporally distant. In this review, we focus on translation, the final stage of gene expression, and examine its coupling with key stages of mRNA metabolism: synthesis, processing, export, and decay. For each of these processes, we provide an overview of known instances of coupling with translation. Furthermore, we discuss the role of high-throughput technologies in uncovering these intricate interactions on a genome-wide scale. Finally, we highlight key challenges and propose future directions to advance our understanding of how coupling mechanisms orchestrate robust and adaptable gene expression programs.

## Introduction

Gene expression is a complex, multi-step process that translates the information encoded in genes into functional products, such as proteins or various non-coding RNA molecules. This process is not merely a linear pathway but involves a series of highly regulated and interconnected stages which allow cells to dynamically adapt to environmental signals, differentiate into specialized cell types, and execute a vast array of cellular functions that sustain life. The regulation of gene expression is crucial for tissue differentiation [1,2] and homoeostasis [3] and occurs at multiple stages – transcriptional, post-transcriptional, translational and post-translational. Dysregulation of these stages is associated with a wide range of diseases, including cancer [4] in which aberrant gene expression patterns can lead to uncontrolled cell growth and metastasis [5]. Over the past decades, the molecular mechanisms underpinning each stage of gene expression have been widely investigated, leading to the discovery of the determinants that govern the RNA lifecycle, including transcription factors, modifications of DNA, chromatin and transcripts, and RNA binding proteins [6].

More recently, a number of studies challenged the simplistic view that gene expression programs result from a linear chain of stages along the RNA lifecycle. Instead, a network of cross-talk mechanisms emerged and established the tight coordination among the various steps of RNA metabolism [7,8]. Important advances shed light on the coordinated regulation between pairs of sequential steps of the RNA lifecycle, such as transcription and processing [9] or translation and degradation [10,11]. More surprisingly, coupling mechanisms have also been identified between steps that are neither sequential nor occurring in the same cellular compartment, as for the case of transcription and translation, suggesting a complex coordination between stages that are significantly temporally and spatially separated. Finally, the power of these far-reaching regulatory mechanisms is demonstrated by their ability to feedback regulatory input from a given RNA lifecycle stage to upstream stages [12].

Within this review, we cover the mechanisms that establish the coupling between mRNA translation – a pivotal stage in gene expression programs – with other primary stages of the mRNA lifecycle: transcription, processing, export, and degradation. We begin by summarizing current knowledge on each of these interactions to clarify how translation influences and responds to other regulatory steps. We discuss the role of high-throughput technologies on the study of coupling mechanisms, and currently available experimental and computational frameworks which can be used to shed light on this phenomenon at the genome-wide level. Finally, we identify challenges in the field and opportunities for future developments which could contribute to advancing our understanding of how mRNA translation integrates into gene expression programs.

## Coupling mRNA translation and transcription

The transcription of genetic information into mRNA and its subsequent translation into proteins are the core processes that drive the expression of coding genes. In bacterial cells, transcription and translation take place in the same cellular compartment, allowing a pioneering ribosome to initiate translation on nascent mRNA while it is still being transcribed by the RNA polymerase complex. This simultaneous engagement on the mRNA strand is known as coupling of transcription and translation [13]. In Escherichia coli, mRNA abundance – closely linked to cell physiology – is positively associated with ribosome occupancy and ribosome density, suggesting the synergic regulation of transcription and translation to reach optimal gene expression regulation [14]. A recent study has further explored this coupling proving that, through mechanical and allosteric effects, the ribosome is able to reduce RNA polymerase pausing and termination thus increasing the rate of transcription at the cost of a loss of fidelity [15]. An additional study has shown that, in situations of translation impairment (e.g. under nitrogen starvation), transcription is slowed down by the action of the stress alarmone (p)ppGpp, restoring the disrupted coordination [16]. Thus, in prokaryotes, transcription and translation are tightly coupled, with specialized complexes maintaining this connection even when one of the two processes is severely disrupted.

Eukaryotic cells feature distinct and specialized compartments, with transcription taking place in the nucleus and translation in the cytoplasm. This physical separation has led to the long-standing belief that these two processes are entirely decoupled [17,18]. Nevertheless, in the last few years, several studies have shown that transcription and translation are coordinated even if spatially separated [19–21]. As an example in yeast, the association of Rbp4 and Rbp7 with RNA polymerase II, necessary for transcription, is followed by the shuttling of the Rbp4/7 heterodimer into the cytoplasm [22–24]. There, Rbp4/7 interacts with the eukaryotic initiation factor 3 complex (eIF3) – a complex of translation factors regulating several steps of mRNA translation, linking transcription with translation initiation [20]. In a different study, it was shown that the binding of Rvb1 and Rvb2 - ATP binding proteins implicated in protein assembly and chromatin remodelling – to the promoter of genes involved in glucose metabolism increases their transcription during glucose starvation. At the same time, the binding of those factors to the mRNAs of the same genes leads them to mRNP granules and reduces their translation [25].

Another example of coupling between transcription and translation, present in both prokaryotes and eukaryotes, is mediated by codon usage. Codon usage is known to regulate the speed of mRNA translation elongation, positively influencing translational efficiency. More recently, however, it has also been identified as a transcriptional regulator. Studies on the filamentous fungus Neurospora have shown that codon optimization correlates with a stronger Pol II density, and consequently enhances mRNA levels. Conversely, non-optimal codon usage is associated with transcriptional repression, mediated by H3K9me3 deposition [26]. Similarly, in mammals, codon optimization in Toll-like receptor 7 (TLR7) mediates both increased transcription – due to higher GC content – and increased protein production. Proper regulation of TLR7 expression is particularly relevant since its imbalance with TLR9 expression can hamper immune surveillance leading to autoimmune diseases [27].

Besides codon usage, Slobodin and colleagues further explored the coupling between transcription and translation in mammals by performing an unbiased genome-wide screen to study the influence of human promoters on mRNA translation. They confirmed that the presence of TATA-box in the promoter enhances transcription while fostering translation [18,28] - the widespread adoption of alternative promoters underscores the potential relevance of this regulatory layer in shaping gene expression [29]. Furthermore, they proposed a mechanism in which co-transcriptional incorporation of the N6-methyladenosine (m6A) RNA modification increases when RNA polymerase II elongation is slowed down. In turn, the higher abundance of m6A mediates the subsequent reduction of those mRNAs translation [18]. Subsequent studies have confirmed the link between RNA methylation and translation [30] suggesting that m6A can both promote and impair protein synthesis, especially during stress responses [31]. Therefore, while m6A represents a promising effector in coupling transcription and translation, further research is required to fully characterize the role of this modification on this coupling.

The existence of so many different factors coupling transcription and translation across different domains of life, both in physiological conditions and in response to stress, highlights the necessity of coordinating these processes to ensure proper cellular function.

## Coupling alternative mRNA processing and translation

Thanks to recent advancements in transcriptomics technologies, including long-read sequencing, we are now able to fully appreciate the diversity of transcripts being expressed in a cell. As a result, we have began appreciating that alternatively processed RNA products from a single gene can drastically differ in their engagement with the translation machinery. These results have been obtained by combining cellular fractionation with RNA sequencing and have shown that alternatively spliced products can be more or less translated [32]. Similarly, different studies employing polysome profiling or Ribo-seq highlighted the importance of considering alternative isoforms when quantifying translation [33–35].

**Figure 1.**
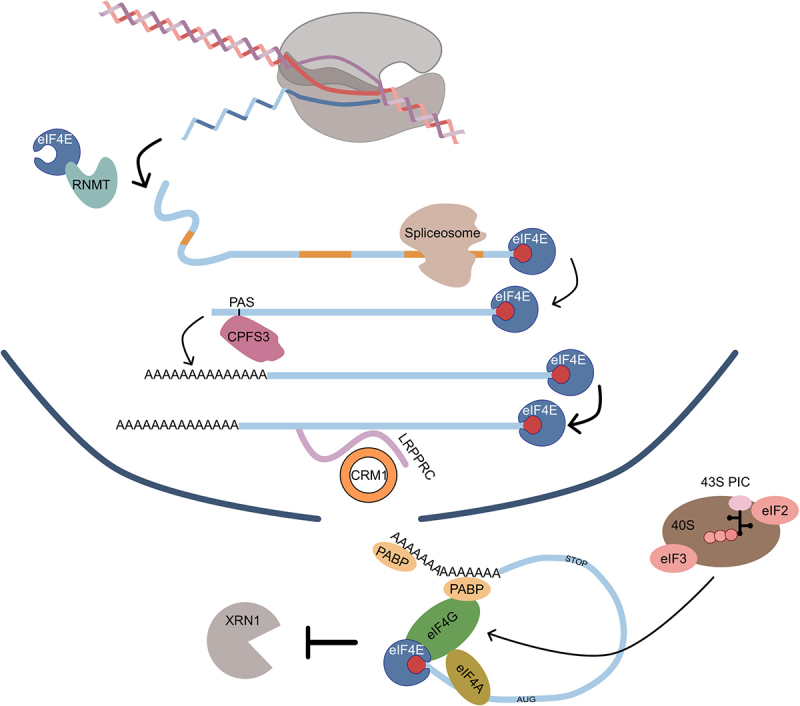
Scheme of the couplings mediated by the translation initiation factor eIF4E. Following mRNA transcription, eIF4E can interact with the capping enzyme RNMT to facilitate capping of mRNAs. Moreover, eIF4E can associate with the spliceosome in a cap-dependent manner and affect its composition, influencing specific splicing events for thousands of genes. Being able to associate with some components of the polyadenylation machinery, e.g. the enzyme CPFS3 which recognizes the polyadenylation sites, eIF4E also plays a role in the 3’ end processing. Furthermore, eIF4E mediates the export of 4ESE mRNAs, containing binding sites for LRPPRC that in turn can bind CRM1, a major export protein. Finally, eIF4E recruits mRNAs to the ribosome to its cap-binding activity and, being associated with the cap, inhibits the action of the decapping enzymes.

While a significant portion of the alternatively spliced transcriptome can generate different protein isoforms, increasing attention is now given to the creation of ‘unproductive’ transcripts by alternative splicing [36]. Intron retention, as well as the inclusion of ‘poison’ exons, can create the presence of an in-frame premature stop codon (PTC), which in turn triggers mRNA degradation via nonsense mediated decay (NMD, which will be explained in more detail in the next sections) [37]. While often impacting non-coding transcripts hosting snoRNAs, fundamental ncRNAs in rRNA maturation [38], NMD also targets transcripts coming from protein-coding genes, often related to splicing, mRNA processing, or translation. One prominent example is EIF4A2, which encodes for a translation initiation factor. EIF4A2 hosts multiple snoRNAs which by interacting with their host transcripts lead to the production of different NMD-sensitive transcripts [39]. At the same time, NMD deficiency can result in the production of EIF4A2 truncated proteins, which increase mTORC1 activity and translation rates and cause differentiation delays [40]. Moreover, many ribosomal protein genes are potentially affected in their processing by hosted snoRNAs. This suggests a complex crosstalk between ribosome biogenesis, translation, snoRNAs and NMD which largely remains to be elucidated. Diversity at the UTR level is fundamental in determining the translation output of an mRNA [41]. For instance, in mammalian cells, the length of 5ʹUTRs was shown to negatively affect translation efficiency due to the regulatory effect of diverse sequence features within these regions [42]. Moreover, the 5’ terminal oligopyrimidine (5’TOP) motif, comprising an invariant 5’-cytidine followed by an uninterrupted tract of 4–14 pyrimidine nucleotides and preceded by the 7-methylguanosine triphosphate, is known to greatly impact the sensitivity to mTOR. Indeed, variations on single nucleotides have significant effects on mTOR-mediated mRNA translation [43]. The large variation of 5’ end sites revealed by large-scale atlases of transcription start site usage for a plethora of cells and tissues [44] suggests that decoding the coupling between transcription initiation and protein synthesis represents an important challenge in functional genomics. Similarly, alternative 3’UTR composition impacts translation, with many 3’UTR binders involved in many aspects of mRNA metabolism, often intertwined. For example, CPEB1 is an RNA binding protein functioning both in the nucleus and the cytoplasm to regulate the expression of mRNAs containing the cytoplasmic polyadenylation (CPE) motif [45]. In the nucleus, CPEB1 can trigger cleavage and polyadenylation at proximal polyadenylation sites, creating highly translated mRNAs with short 3’UTRs [46]. In the cytosol, CPEB1 binds RNA but to stimulate polyadenylation, thus promoting both translation and mRNA stabilization [45]. Exemplifying the crosstalk between RNA processing and translation is the finding that SRSF1, a fundamental factor regulating pre-mRNA splicing, also promotes translation initiation by inhibiting eIF4E-binding proteins, translation repressors regulating the activity of the translation factor eIF4E. This creates a potentially direct link between splicing and translation initiation regulation [47,48]. Noticeably, eIF4E also regulates the export of mRNAs encoding for several splicing factors, thus influencing the expression of different alternative transcripts [49]. This also applies to key components of the capping machinery, such as RNMT, which is enhanced in expression and translation by eIF4E and is one of its direct interactors [50]. In addition, eIF4E physically interacts with components of the cleavage and polyadenylation complex, like CPSF3 [50,51], further supporting its role as a fundamental mRNA regulator, from nuclear processing to export and translation initiation (Figure 1).

RNA modifications have also been linked to mRNA processing and proposed as coupling factors. m6A has been reported mediating splicing regulation through the action of reader proteins such as YTHDC1, HNRNPC, HNRNPG, and HNRNA2B1 [52–55]. Furthermore, a mechanism involving competition between the exon junction complex and the m6A writing machinery has been recently proposed to explain the mRNA methylation landscape [56]. These findings suggest a role for this mark in mediating the coupling between mRNA splicing and translation, facilitated by the aforementioned m6A-dependent modulation of the latter process.

With increasing mechanisms of mRNA diversification, including alternative splicing, modification and polyadenylation, the ensemble of regulatory feedbacks between the complex world of mRNA processing and tight control of protein synthesis is only starting to be unveiled.

## Coupling mRNA translation and export of transcripts to the cytoplasm

As previously mentioned, eukaryotic cells differ from prokaryotic ones for the presence of the nucleus which spatially separates the synthesis and processing of mRNAs from their translation into proteins. This segregation is functional to the implementation of mRNA control mechanisms: in the nucleus, premature mRNAs are processed into mature transcripts, undergoing quality controls before being exported and translated into proteins. The fact that processing and translation happen in two different compartments prevents translation machinery from operating on immature mRNAs that are not properly processed.

The efficiency of mRNA export depends on key features, the most important being a correct splicing and a high GC-content at the 5’ end of mRNA, which also enhances mRNA translation efficiency [57]. Export has also emerged as a regulatory mechanism that actively impacts protein abundance by prioritizing the export of selected mRNAs [58,59].

Not surprisingly, numerous examples exist where the impairment in mRNA export negatively impinges on the translation of those transcripts. The germinal centre-associated nuclear protein (GANP) has been shown to play an important role in export by binding both the nuclear pore complex and NXF1. Specifically, GANP promotes the nuclear export of mammalian mRNAs which are bound by the NXF1 export factor. In yeast, it has been found that this specific set of mRNAs codes for proteins with increased translational efficiency, high protein stability and high protein abundance [60]. The coupling between export and translation operated through the N×F1export pathway is also relevant for the Werner syndrome, which causes premature ageing due to a single mutation on a chr8 gene coding for the WRN Werner syndrome protein. WRN depletion has a negative effect on protein synthesis, which is not due to a stress response or reduced number of ribosomes, but arises through reduced export in WRN-depleted cells mediated by the disrupted interaction of this protein with NXF1, causing an imbalance in the mRNAs subcellular distribution [61]. In another pathological context, a study conducted on alpha-synuclein (SNCA) protein, a key player in Parkinson’s disease, identified the heterogeneous nuclear ribonucleoprotein D (HNRNPD or AUF1) as the responsible for SNCA mRNA export impairment, impacting on translation and thus on protein levels [62].

In all the aforementioned examples, the export factors do not interact directly with the translational machinery, and therefore represent indirect examples of coupling. As a direct evidence of the coordination between export and translation, studies in S. cerevisiae revealed that the export factor Gle1 (yGle1) plays a role both in translation initiation, where it interacts with the eukaryotic translation initiation factor eIF3 modulating the function of the DEAD-box helicase Ded1, and in translation termination, where together with the inositol hexakisphosphate (IP6) regulates Dbp5 [63]. A role of Gle1 in regulating both export and translation has also been documented in human where hGle1 plays a role in the distribution of mRNPs between stress granules and translation by its regulation of the translation initiation factor DDX3 [64]. Another relevant example of coupling between export and translation is operated by the eukaryotic translation initiation factor eIF4E. This protein is found both in the nucleus, where it binds the caps of eIF4E-sensitive element (4ESE)-containing RNAs, and in the cytoplasm, where it interacts with capped RNAs and positively impacts the translation of 30–50% of them [50,65]. eIF4E mediates the export of its targets by interacting, thanks to the aid of leucine-rich pentatricopeptide repeat C-terminus protein (LRPPRC), with CRM1, a key component of a major export pathway that serves as an alternative to the bulk NXF1 pathway [50]. Notably, treating cells with the CRM1 inhibitor Leptomycin B reduces the export of 4ESE-containing mRNAs but does not affect bulk mRNA export [50] (Figure 1).

Finally, m6A has been suggested as a potential coupling factor between mRNA export and translation, owing to its impact on protein synthesis and the role of the nuclear reader YTHDC1 in promoting mRNA export [66].

## Coupling mRNA translation and degradation

The degradation of mRNA molecules is a key step in gene expression, serving three main functions: ensuring quality control to prevent the translation of aberrant transcripts, defining steady-state mRNA expression levels, and modulating mRNA responsiveness to stimuli – all of which are crucial for determining the abundance of functional proteins [67,68]. In this section, we cover the coordination between translation and mRNA decay.

In E. coli, mRNA synthesis, translation and decay are directly coordinated. It has been shown that inhibition or impairments in translation initiation and inhibition in translation termination lead to an accelerated decay [69], while impairments in the rate of translation elongation can lead either to transcripts stabilization or to faster decay [70].

In eukaryotes, most mRNAs are degraded via a canonical cytoplasmic pathway that begins with transcript deadenylation. This step is followed by either degradation of the molecule from its 3’end or decapping of the transcript and subsequent decay from its 5’ end [71]. The presence and length of the poly(A) tail are crucial for regulating mRNA stability. Typically, mRNA poly(A) tails are initially synthesized with an average length of 150–250 nucleotides [72] and are dynamically regulated in the cytoplasm [73]. Their distinctive homopolymeric structure allows them to be fully coated by sequence-specific poly(A)-binding proteins, primarily PABPN1 and PABPC1. PABPN1 resides in the nucleus, where it contributes to poly(A) tail synthesis by stimulating its extension [74] and facilitating efficient splicing of the last intron of transcripts [75]. PABPC1, by contrast, functions in the cytoplasm, where it regulates mRNA decay and translation, serving as a key coupling factor between these two steps of gene expression [76]. PABPC1 interacts with the cap-binding complex via eukaryotic initiation factor 4 G (eIF4G) to establish a closed-loop configuration that enhances translation efficiency [77]. In this regard, in vitro replication of the cap-poly(A) synergy has shown that PABPC1 facilitates translation on poly(A)-containing mRNAs in a cap-dependent manner, while mutations impacting the interaction between PABPC1 and eIF4G leads to a significant decrease in translation levels [78]. Furthermore, PABPC1 is involved in translation termination by interacting with release factors, thereby facilitating efficient recycling of ribosomes [79]. PABPC1 has also been shown to interplay with other RNA-binding proteins like DEAD-box helicases, which influence mRNA structure and accessibility during translation [80]. Finally, the interaction of PABPC1 with the 5’ cap structure is proposed to stabilize eIF4E [81] and sterically hinder the binding of decapping enzymes, thereby reducing mRNA susceptibility to degradation. In this regard, the association of PABPC1 with the poly(A) tail can also protect the 3’ end of mRNA molecules, preventing exonucleases from accessing it [82]. Indeed, experimental evidence suggests that deadenylation requires the dissociation of PABPC1 from the poly(A) tail. In vitro studies further demonstrate that increasing PABPC1 levels can inhibit deadenylation, while sequestering PABPC1 exposes mRNA to destabilization [82]. While the importance of poly(A) tails for the coordination of mRNA decay and translation is established, the full picture is remarkably complex. For instance, the role of poly(A) tails in this coupling seems to vary along with developmental stages. Studies on maturing oocytes and early embryoshave shown that the lengthening of poly(A) tails is essential for regulating gene expression during the early stages of animal development [83]. As expected, longer poly(A) tails enhance translation efficiency, facilitating robust protein synthesis during early development [84]. However, as development progresses, this coupling diminishes, and poly(A) tail length no longer significantly impacts translation. Furthermore, Xiang and Bartel examined frog eggs and early embryos, where PABPC1 and poly(A) tails primarily promoted the translation of mRNAs without significantly affecting their stability. In contrast, at later developmental stages, these factors contributed more to mRNA stability than to translation [76].

Other relevant examples of coupling between the mRNA canonical decay pathway and transcript translation involve RBPs and miRNAs. For instance, the RBP Regnase-1 associates with polysomes and destabilizes transcripts in a translation-dependent manner [85]. Similarly, miRNAs mediate a dynamic crosstalk between mRNA translation and degradation through their role in the RNA-induced silencing complex (RISC). Guided by miRNA base-pairing, RISC targets specific mRNAs and recruits GW182, a key mediator of downstream silencing mechanisms. GW182 disrupts PABPC1 activity, reducing translation efficiency, and sensitizes mRNAs to deadenylation via recruitment of the CCR4–NOT complex [86]. While this mode of action is well established, many examples deviate from this model, suggesting a more complex scenario that warrants further exploration [87].

Despite the existence of mechanisms to ensure the quality of mRNAs produced and processed in the nucleus [88], defective mRNAs might reach the cytoplasm. Various quality control pathways are indeed intrinsically associated with translation in this compartment, including non-stop decay (NSD), no-go decay (NGD) and nonsense mediated decay (NMD). NSD targets mRNAs lacking a termination codon and is activated when ribosomes fail to recognize a stop codon during translation, thus proceeding until the 3’ end and occupying the A site. NGD, instead, happens in case of stalling events in translation elongation, leading to endonucleolytic cleavage of mRNA and exonucleolytic cleavage of the 5’ and 3’ ends. NMD targets mRNAs containing a premature termination codon (PTC), placed anywhere upstream the stop codon, originated from genes harbouring nonsense or frameshift mutations. One of the two pathways for NMD activation involves the competition of the core UPFs proteins with PABPC1 for the recruitment of the translation termination complex: if UPFs are faster than PABPC1 NMD is triggered, otherwise translation termination leads to a new cycle of translation [89].

Finally, the m6A RNA modification is not only important for the coupling of translation with transcription, processing and export, but also for coordinating mRNA translation to decay. Indeed, this modification is not only involved in translation regulation [30,90] but also prominently involved in the regulation of transcripts half-lives. In this context, the readers YTHDC1, YTHDC2, and YTHDF1, YTHDF2, YTHDF3, are known to promote decay [91–94]. Specifically, YTHDF2 acts partially through the recruitment of the CCR4-NOT deadenylase complex [95], and the conservation of its region interacting with CNOT1 in YTHDF1 and YTHDF3 suggests a similar mechanism for these two effectors [96]. A recent study suggested the involvement of YTHDF2 in a novel decay pathway triggered by m6A-dependent ribosome stalling [97]. Intriguingly, the IGF2BP1–3 m6A readers have been shown to act in the opposite direction, stabilizing target transcripts [98].

## Omics data and computational approaches for the study of coupling mechanisms

The study of specific regulatory networks, along with the global exploration of transcriptional and translational programs, revealed a broad and complex coupling between translation and various stages of the mRNA lifecycle. Bioinformatic approaches can aid researchers in unravelling this complexity, whether for small detailed regulatory networks, or at a genome-wide scale through simpler but more general models. In the first scenario, dependencies between translation and other steps of the RNA lifecycle can be hard-coded into equations modelling the system of interest, see for instance [99] for examples on autoregulatory genetic feedback loops. This allows for in-depth characterization of specific instances of coordination between steps in gene expression. Conversely, the second approach is more exploratory and better suited to studying coupling mechanisms emerging from data that involve a significant number of genes, albeit with less detail on the underlying mechanism. This section of the review will focus on the latter paradigm.

The genome-wide exploration of the interplay between the mRNA and protein lifecycles has been enabled by remarkable technological advancements over the past decades. These developments have made it possible to generate transcriptional, translational and proteomic maps with reasonable cost and effort. On one hand, mass spectrometry (MS) based approaches, when coupled with internal standards, allowed quantifying the absolute expression for thousands of proteins [100,101]. Similarly, high-throughput RNA sequencing platforms can quantitatively measure the abundance of tens of thousands of transcriptional units when accompanied by the use of spike-ins [102]. Notably, the same RNA-seq technologies when applied to mRNA associated with ribosomes can also provide a proxy for the efficiency of protein production assuming the same rate of elongation for all transcripts [103,104].

The application of high-throughput platforms has primarily facilitated the study of correlations between mRNA and protein expression levels. These correlations have been examined across-genes within the same biological sample and for individual genes across-samples [105]. Such studies have begun to shed light on the simplest form of coupling between transcriptional and translational programs. Wang and colleagues explored this aspect using a dataset of 29 human tissues, reporting correlations ranging from 0.42 to 0.57 across genes and a median correlation of 0.35 across samples with 90% of correlations being positive and half statistically significant [106]. This reduction in correlation across samples can be partially attributed to increased noise when multiple samples are involved, as well as the smaller variability in mRNA and protein expression levels across samples compared to across genes. On the other hand, this observation may suggest that the primary role of transcriptional regulation is defining the overall magnitude of protein expression, while translational regulation is primarily responsible for setting condition-specific protein expression levels [107]. Despite these modest while significant correlations, how well mRNA expression levels recapitulate protein abundances still remains an open question subject of significant debate.

These types of comparisons can also be conducted for specific RNA pools. For instance, Slobodin and colleagues profiled both the sites of active RNA polymerase II using GRO-seq and ribosome occupancy with Ribo-seq in human cell lines. Using these datasets as a proxy for transcriptional and translational activity, respectively, they observed modest yet significant correlations between the two processes (0.15–0.22) [18]. Similarly, Schott and colleagues combined RNA metabolic labelling with Ribo-seq to focus on newly transcribed mRNAs, revealing that mRNA decay and ribosome loading are positively correlated, which could enable the translation of unstable transcripts before their decay [108]. Compared to the profiling of nascent transcription through GRO-seq (or RNA metabolic labelling), the development of nascent Ribo-seq also has the advantage of not being complicated by the temporal lag between transcription and translation. The coordination of transcriptional and translation responses over time has been studied by collecting data over time in response to stimuli, thus requiring more sophisticated approaches that move beyond the steady-state perspective [109]. Such methodologies, which are essential for studying transient responses, can also contribute to resolving the complexity of mRNA and protein lifecycles at steady state.

A substantial body of literature is now available on the modelling of the mRNA lifecycle. The general framework typically involves a system of Ordinary Differential Equations (ODEs) used to describe the temporal dynamics of transcript abundance for a given transcriptional unit. The parameters of these equations, known as kinetic rates, have clear biological interpretations and reflect the efficiencies of specific biological processes included in the model (e.g. mRNA synthesis, splicing, export, decay, association with polysomes, etc.). Once the mathematical framework is defined, RNA species abundances are quantified to infer the values of these kinetic rates.

Frameworks proposed in the literature primarily differ in the aspects of the RNA lifecycle they represent and, consequently, in the amount of information required for inference. Importantly, to estimate absolute kinetic rates, all models necessitate data that capture the temporal dynamics of RNA species. For example, RNA-seq time-course data collected in response to a stimulus were used to infer rates of mRNA synthesis and degradation over time [110], and were subsequently extended to include RNA processing [111]. Alternatively, other studies captured the temporal dynamics by profiling nascent transcription through RNA metabolic labelling with nucleoside analogues (commonly 4sU or 5eU) [112,113]. Leveraging these powerful frameworks, the field has progressed from the study of simple models of the RNA lifecycle [113] to more complex representations accounting for mRNA trafficking across cellular compartments [114–116] and its association with polysomes [114,115]. Focusing on the latter aspect, Letswaart and colleagues quantified the flow of mRNA across compartments and observed that a subset of genes involved in stimulus responses exhibited fast kinetics which suggested global regulation of all rates, including polysome association. However, they found no correlation between cytoplasmic turnover and polysome loading; the two processes competing for cytoplasmic mRNA in their model [114]. An independent study corroborated this last observation reporting weak yet significant negative correlations between the rate of polysomal association and both mRNA synthesis and polysomal mRNA degradation [115].

The general modelling framework described above also largely applies to the study of the protein lifecycle. However, in this case, protein abundances profiled by MS and directly modelled in the ODEs must be complemented with RNA-seq data, as transcripts are directly involved in translation. In this context, modelling efforts have primarily focused on estimating rates of protein synthesis and degradation. In a pioneering study, Tchourine and colleagues analysed three datasets that reported protein and mRNA expression levels over time in yeast responding to different stimuli, and they successfully estimated constant kinetic rates for approximately 30% of the profiled genes. Interestingly, their findings indicated that protein synthesis and degradation rates were largely uncorrelated [117]. However, they also observed that for most of the genes one rate could vary widely without significantly impacting the goodness of fit of the model; a scenario that questions the result of these correlative studies. More recently, van den Berg and colleagues applied the same paradigm to study a 96-hour differentiation time-course [118]. By fitting this dataset with models of varying complexity, they demonstrated how dynamic modelling can effectively integrate multi-omics data to dissect gene expression programs. Alternatively, several studies have utilized heavy stable-isotope metabolic labelling (pSILAC) to profile the nascent proteome. This approach allows for the precise estimation of synthesis and degradation rates, thereby enabling a more complete resolution of the system [119].

Despite remarkable advancements in studying mRNA and protein lifecycles, relatively few studies have explored the interplay between stages of these two processes. In a seminal study, Schwanhäusser and colleagues determined mRNA and protein synthesis and degradation rates at steady-state in mouse fibroblasts [120], revealing that genes with specific combinations of mRNA and protein half-lives often share common functions. For instance, genes required for rapid responses to stimuli, such as transcription factors and cell cycle-specific genes, were characterized by unstable mRNAs and proteins, likely enabling rapid transcriptional and translational regulation. However, they did not find a statistically significant global correlation between transcript and protein half-lives. Schwanhäusser et al. did not analyse the relationship between mRNA and protein synthesis rates, but they focused on the latter which they identified as the best predictor of translational outcomes. This finding was later questioned in a reanalysis of their dataset [121], which emphasized the role of mRNA expression and kinetic rates in determining protein abundance but did not provide additional insights into correlations between kinetic rates. Further investigation of this latter aspect would have been particularly relevant, as recent work reported a significant correlation between mRNA and protein half-lives in yeast [122]. This finding disagrees with the conclusions of Schwanhäusser and colleagues, and the discrepancy could stem from bona fide biological differences, but also from data analysis issues. In 2014, Rabani and colleagues investigated the temporal modulation of mRNA synthesis, processing, and degradation rates during the lipopolysaccharide (LPS) response in mouse dendritic cells. For a subset of genes characterized by transcriptional repression and reduced degradation rates, they also observed increased translation rates [68]. In a subsequent study, the same group conducted nascent and total proteomics profiling to investigate this system more deeply. They found that mRNA modulation, which was primarily driven by transcriptional regulation, dominates protein changes. Additionally, protein synthesis and decay rates were shown to contribute similarly to translational modulation [123].

Altogether, omics approaches, aided by mathematical modelling, have revolutionized our capacity to investigate coupling mechanisms between the mRNA life-cycle and translation on a genome-wide scale. While the findings strongly support the existence of an extensive coupling network, the overall picture remains controversial. Further application and development of these tools are essential to deepen our understanding of these coordinations. In the next section of this manuscript, we outline potential new avenues of exploration for this field.

## Perspectives

**Figure 2.**
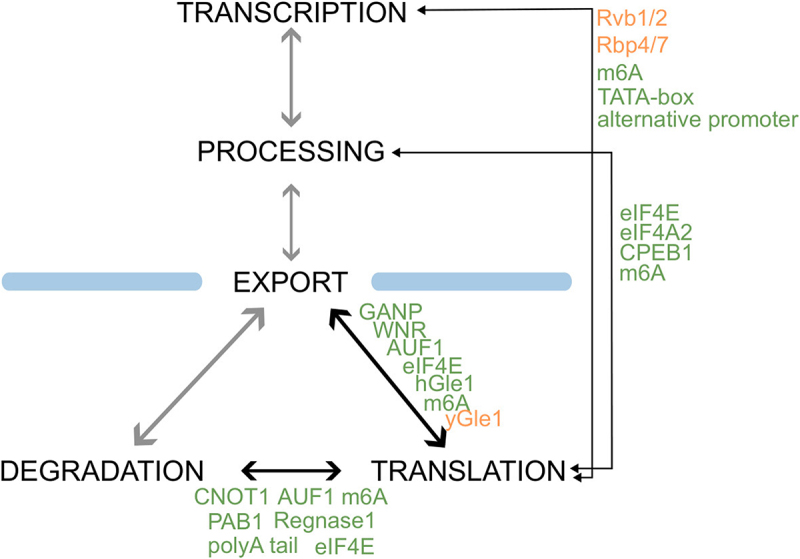
Scheme of the coupling mechanisms: Ticker arrows represent natural couplings, depicted in black if they involve translation and in grey otherwise. Thinner arrows indicate translation-related long-range couplings. Each edge lists the factors mediating couplings in eukaryotes (green) and yeast (orange).

This review underscores the existence of an intricate network of mechanisms, mediated by a diverse array of effectors, that orchestrate a coordinated modulation of translation with key steps of the mRNA lifecycle (Figure 2). While a growing body of evidence supports the existence and relevance of these mechanisms, it is likely that we have only started revealing the full complexity and implications of this coordination. Numerous avenues of investigation remain and others will open.

First, attention should be paid to translation occurring in cellular compartments other than the cytoplasm. For example, the translation of most mitochondrial mRNAs is known to be coupled with their processing and stability through the action of the LRPPRC/SLIRP complex [124]. Several other RNA-binding proteins involved in translation and other steps of mitochondrial gene expression have been identified (e.g. GRSF1, MTRES1, MRPL12, and FASTKD3), suggesting that a more complex coupling network remains to be dissected [124]. Mitochondrial non-coding RNAs have the potential to impact the stability and translation of their coding targets, but their role as coupling factors is largely unknown [124]. A second example of non-cytoplasmic protein synthesis is the controversial occurrence of translation in the nucleus, an intriguing process to investigate, as it may reveal novel coupling mechanisms with canonical nuclear processes, such as transcription; a concept which nicely traces back to prokaryotic biology [125].

The analysis of the dynamics of mRNA deadenylation has recently expanded by using state-of-the-art techniques like poly(A) tail quantification by sequencing, nascent RNA profiling, cellular fractionation, and mathematical modelling. This approach revealed rapid nuclear deadenylation as a widespread mechanism in humans and mice, producing gene-specific poly(A) tail lengths prior to export. This represents a regulatory layer that could significantly expand poly(A) tail-mediated couplings [126]. In this regard, the length of poly(A) tails in chromatin-associated transcripts has been shown to be reduced in response to splicing inhibition, concomitant with a reduced rate of export of those mRNAs [115]. Efforts to modify poly(A) tails have opened promising avenues for improving protein yield from mRNA templates. Chen et al. recently demonstrated that engineered and chemically modified poly(A) tails provide a more robust binding platform for poly(A)-binding proteins, thereby reinforcing the mRNA’s closed-loop configuration and boosting translation efficiency [127]. Such tailored enhancements not only advance our understanding of the molecular mechanisms governing gene expression, but also hold profound implications for the future of mRNA-based therapeutics.

Third, we believe attention should be directed towards studying coupling mechanisms that propagate information in the opposite direction to canonical gene expression. An intriguing possibility involves competition for coupling factors, where their sequestration in downstream events could indirectly impact upstream processes.

Fourth, we highlight the importance of investigating the crosstalk between translation and other stages of the RNA life cycle in the context of cell metabolism. Indeed, gene expression is an extremely energy-intensive process; therefore, it must be tightly regulated and optimized in response to growth and nutritional conditions – a regulation that is likely to rely on coupling mechanisms [128].

Finally, we would like to remark the importance of expanding our omics-driven computational toolkits to ease the study of gene expression coupling mechanisms. In terms of modelling, we anticipate the need to fully integrate RNA and protein lifecycles into unified computational frameworks. This effort could also be directed towards resolving specific stages, such as transcription and translation, into the composing steps, such as RNA polymerase II [129] or translation elongation [130]. Such improvement would likely reveal novel coupling mechanisms between specific steps of the translation cycle, and among these and other stages of gene expression. These approaches should leverage models and model selection theory to test direct coupling hypotheses. This would allow going beyond simple correlative analyses, offering more mechanistic insights and enabling the identification of genes specifically subject to specific coupling mechanisms. This latter aspect would be particularly important since correlative analyses tend to overlook coupling instances involving a small set of transcriptional units which, however, could be biologically relevant.

Another critical computational area for exploration is moving beyond steady-state comparisons to investigate the modulation of kinetic rates over time, an approach already applied to simple models of the mRNA lifecycle. This would reveal whether couplings shape the transient modulation of the rates rather than their steady-state values. This would also inform on the causality and directionality of coupling mechanisms – e.g. given two coupled rates, which one is directly affected and responds first to a perturbation. While any treatment is suitable for these analyses, perturbations directed at specific steps of gene expression would be simpler to interpret. For example, the coupling between two stages could be tested by directly perturbing the first one and checking the consequence on the second one. The perturbation (e.g. knock-down) of putative coupling factors would be better suited to confirm their role as coupling mediators. Rather, it would provide an exceedingly complex readout regarding the existence of the coupling itself. A complementary alternative to test how a given coupling is mediated by a putative coupling factor is the use of transgenes that carry regulatory elements responsible for the recruitment of the coupling factor [131].

## Conclusions

We discussed several examples of coordination occurring between translation and key steps of the mRNA lifecycle, and we revealed the numerous effectors responsible for their establishment. We also provided an overview of the omics frameworks which can be exploited to study these coupling mechanisms, and how mathematical modelling can enhance the interpretation of similar datasets providing important insights otherwise hidden in data convolution. Finally, we suggested several directions to follow which, in our opinion, could contribute to the evolution of this research field. This manuscript represents a first contribution in this direction, which we hope will be complemented by other works going beyond the translation-centric perspective.

## Data Availability

Not applicable, as no datasets were used in the current study.
